# Geochemistry and mineralogy of auriferous tailings deposits and their potential for reuse in Nova Lima Region, Brazil

**DOI:** 10.1038/s41598-023-31133-6

**Published:** 2023-03-16

**Authors:** Mariana Lemos, Teresa Valente, Paula Marinho Reis, Rita Fonseca, João Paulo Pantaleão, Fernanda Guabiroba, José Gregorio Filho, Marcus Magalhães, Bruno Afonseca, Antonio Roberto Silva, Itamar Delbem

**Affiliations:** 1 Institute of Earth Sciences, Pole of University of Minho, Campus of Gualtar, 4710-057 Braga, Portugal; 2 Anglogold Ashanti, Nova Lima, 34000-000 Brazil; 3 Institute of Earth Sciences, Pole of University of Évora, University of Évora, 7000 Évora, Portugal; 4 Microscopy Center, Universidade Federal de Minas Gerais, Belo Horizonte, 31270-013 Brazil

**Keywords:** Environmental chemistry, Environmental impact, Engineering, Natural hazards

## Abstract

Since the mid-nineteenth century, gold ores, mainly hosted in sulfides, have been processed at metallurgical plants located in Nova Lima, Brazil. The generated wastes have been accumulated over the years in tailings dams or in piles. These materials represent wasted from old circuits, as well as from plants still in production. In this study, geochemical, mineralogical, 3D modelling, and metallurgical analyses wastes were carried out to evaluate potential reuse of these wastes. The performed characterization detected residues of very fine grain size containing sulfides and oxides. The wastes show high grades of Au hosted in different minerals. In addition to Au, samples contain S, Fe, Zn, Pb, Sc, Si, and As. The 3D modelling for spatial definition of Au was performed using ordinary kriging with dimensional variograms. The results indicated the occurrence of Au enrichment zones and allowed to reveal the most attractive tailing deposits in terms of Au content. Metallurgical tests showed recovery of 70% of Au and suggested other potential reuse of the wastes, such as aggregates for the civil construction sector and recovery of other metals. The present work highlights the importance of an integrative characterization within the scope of the circular economy and the value of tailings in the production chain of the mineral sector.

## Introduction

In Brazil, the mining sector is one of the most economically and socially important. This sector currently represents between 3 and 5% of the Gross Domestic Product (GDP). The importance of this economic sector goes back to the seventeenth century, at the beginning of the first gold cycle^1^. In the mid-eighteenth century, Brazil produced approximately half of the world's gold, attracting the immigration of around 400,000 Portuguese, mainly to the Nova Lima region, Minas Gerais.

Currently, this sector moves others, such as construction, automotive and aerospace industries, among others, through the supply of raw materials^2^. Although its importance is relevant and strategic for the economy, the generation of abundant tailings materials remains an obstacle to sustainable development. The total inventory of mining wastes in the State of Minas Gerais exceeds 500 million tons^3,4^. The main infrastructures used for the deposition of these wastes are dams and mine dumps that exceed 500 registered occurrences in the state, with at least 15 referring to gold mining^5^. Dams and mine dumps represent risks to the environment and the population, as demonstrated by tragedies of dam collapses in Brumadinho and Mariana regions, both in Minas Gerais, Brazil^6^.

The generation of mining waste associated with possible interruptions in supply and limited replacement, and therefore interruptions in the supply of critical substances, can generate negatives for all parties directly and indirectly involved, with generation of socioeconomic impacts globally^7^. However, these wastes may be regarded as mineral deposits with grades and physical characteristics different from the exploited primary ore^8,9^. Often, mine wastes have interesting concentrations of substances that are currently critical for technological society. This criticality led the EU to create the Critical Raw Materials list^10^. This list includes key elements with economic importance and supply risk. In addition, materials with continuous use and high demand are also highlighted, such as Au^11^. Thus, the need for detailed and in-depth studies on these types of waste is indisputable.

Different approaches have been explored by researchers around the world to address the problem of solid mining tailings generation. There are some paths that aim to balance the exposure of tailings in balance with the environmental, social and economic sectors. These include reuse of waste with its recycling and reprocessing in various ways, leading to proactive waste management. These models aim to add new value to tailings by identifying the proper destination of potentially hazardous materials^12^

Studies using a combination of characterization techniques are the starting point to add value and increased knowledge to these wastes^13^. The integration of analytical techniques such as specific chemical analysis for solids, mineralogical and particle size distribution, and density determination, can be found in a number of publications^6,13–19^ (demonstrating that this characterization stage is critical to evaluating and understanding the waste deposits.

The use of mathematical and geostatistical tools, common in the numerical definition of mineral deposits, is also of great value in defining the sampling grid, modeling the distribution of grades, tonnage and contaminants dispersion patterns of tailings^20^. Different case studies using mathematical techniques are reported in the literature [e.g.,^13,17,21–23^]. In general, they combine waste characterization and statistical tools to obtain models for the valorization potential while attaining a better understanding of the environmental impacts of the mine dumps.

With such evolutions in the characterization stage, market and technology changes, these wastes can thus provide an alternative to primary exploitation, contributing to the sustainable development of the mining sector and the region. A reuse study based on statistical variables and waste characterization is an important step in the valuation and knowledge of the economic potential of these secondary deposits. This phase covers the processing of any form and types of waste, passing through waste from solid to gaseous phases^24,25^. Various types of proposals can be described in this stage. The reuse of base elements from solutions of acidic drainage in abandoned mining waste deposits^26–32^, the recovery of metals from dam water, and the reuse of solid tailings with recovery of valuable and critical elements for society^33–37^ are some examples of these approaches. Regarding the tailings deposits resulting from the treatment of Au ores^38,39^ proposed different methodologies for recovering this element from secondary sources. The combination of geochemical and mineralogical characterization techniques with hydrometallurgy, pyrometallurgy and even biological agents seems to be effective for Au recovery from tailings deposits.

In addition to these reviews and methodologies, the state-of-the-art shows success in recovering gold from mining tailings through different procedures^6,34,40–42^. Nevertheless, dams and tailings deposits have environmental impact due the potentially hazardous substances they contain^43^. In other words, the economic potential of the huge volume of wastes and the high environmental impact and risk to human health are two opposing and challenging components that justify the interest and the opportunity of the present study.

The present work, therefore, combines the characterization stage with statistical modeling to identify the potential for the valorization of tailings from Au processing in the Nova Lima region-MG, Brazil. The developed methodology is applied in five different deposits in varying stages of use, deposition methods and origin of the tailings. The main aims are (1) to present a characterization of the solid tailings; (2) making comparisons between the different deposits; (3) investigate chemical associations and spatial patterns distribution of the metal contents to assess the potential for metallurgical recovery, especially Au, in the tailings. This work demonstrates the importance of the characterization and processing stages for correct waste management, avoiding socio-environmental impacts and tragedies. Also, it highlights the need to rethinking tailings as potential new products, which further contribute to the sustainable development of the mining sector.

## Local setting

**Figure 1:**
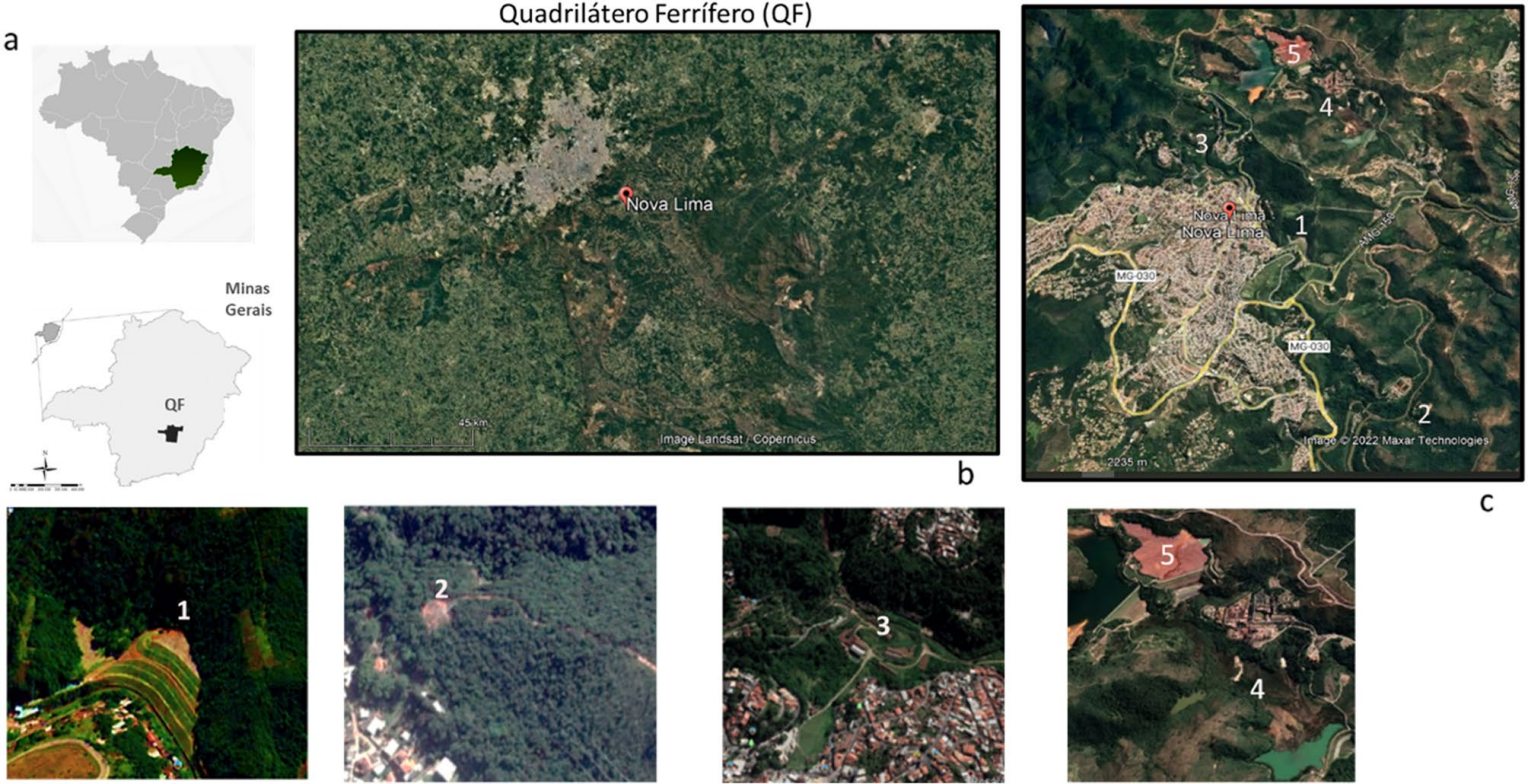
Location of the QF (**a**), Nova Lima Region and the location in the QF (**b**), and the illustration location of the tailings deposit 1 to deposit 5. Google earth Pro V 7.3.6.9345 (64-bit). Image date 6/22/2022 19° 59′ 05.66″ S and 43° 50′ 49.070″ W. The dams and mine dumps in this region are located between the cities of Nova Lima and Raposos, 25 km from its capital, Belo Horizonte, Minas Gerais, Brazil (**c**).

The study areas are in the Quadrilátero Ferrífero (QF), which contains present among the largest deposits of gold and iron in Brazil (Fig. 1a)^44^. Two main tectonostratigraphic domains of Archean and Proterozoic ages, define the QF province: granitic–gneissic terrains, a greenstone belt-like sequence (Rio das Velhas Supergroup) and a supracrustal sequence of chemical and clastic sedimentary rocks (Minas Supergroup) (Almeida, 1967). The first domain, which contains the Rio das Velhas Greenstone Belt, includes the most important gold-bearing region in Brazil^45,46^. The reserves represent 4.5% (936 t) of Au ore worldwide. The deposits are structurally associated and controlled by hydrothermal alteration. In addition, they are hosted in tholeiitic mafic volcanic rocks, banded iron formations, komatiites, metavolcanoclastic shales and phyllites and terrestrial clastic sequences, metamorphosed from greenschist to amphibolite facies^47–49^.

The mines in the Nova Lima region (Fig. 1b) are orogenic gold mineral deposits, as well as others present in the QF, including Morro Velho, São Bento, Raposos, Juca Vieira and Cuiabá, directly related to the evolution of the Rio das Velhas greenstone belt (ca. 2.75 Ga) and its banded iron formations (BIF)^50,51^. The mineralization is a sulfide-enriched BIF. The presence of pyrite (± arsenical) associated with arsenopyrite indicates gold mineralization. In addition, gold is also associated with quartz veins and ranges from weakly to strongly refractory ore.

According to the Koppen classification (humid subtropical climate), the region has a warm and temperate climate. Precipitation is higher in December, with an average of 302 mm and August is the driest month. January is the hottest month of the year and June the coldest, with an average temperature of 17.6 °C^52^.

Deposits 1, 2 and 3 (Fig. 1c) received numerous types of rejects from old gold plants in the region. Unlike deposits 4 and 5, these are over 80 years old deposits that are not currently used. Deposits 1 and 3 are represented by piles, while deposit 2 consists of a dam. For these deposits, it is estimated that the oldest waste from twentieth century may represent gold extraction processes prior to the use of cyanide, which hinders describing the extraction process accurately^53^. Table 1 presents a tentative historical description of the beneficiation processes that have occurred in the oldest deposits, contents of gold (Au), sulfur (S) and arsenic (As), as well as the recovery of Au.

**Table 1:** Forms of Au extraction, mass estimates and concentrations of Au, As, and S for deposits 1, 2, and 3 (modified from^53^).

Area	Operation year	Au recovery (%)	Metallurgical methodology	Tonnage (kT)	Au (mg/kg)	As (%)	S (%)
1	1930–1945	90	Gravity + flotation + CN + Zn precipitation	500	4.93	8.07	9.06
2	1960–1980	93	Gravity + flotation + CN + Zn precipitation	240	1.83	3.7	7.63
3	1834–1930	50–80	Gravity + CN + Zn precipitation	315	4.06	8.87	6.61

At deposit 3 (Fig. 1c—the first plant operating in the city of Nova Lima), tailings from gravimetric recovery (50% Au recovery), cyanide leaching, and Zn precipitation processes have been deposited since the nineteenth century. The wastes in deposits 1 and 2 underwent flotation processes in addition to gravimetry, flotation, cyanide leaching and precipitation using Zn (Table 1).

**Figure 2:**
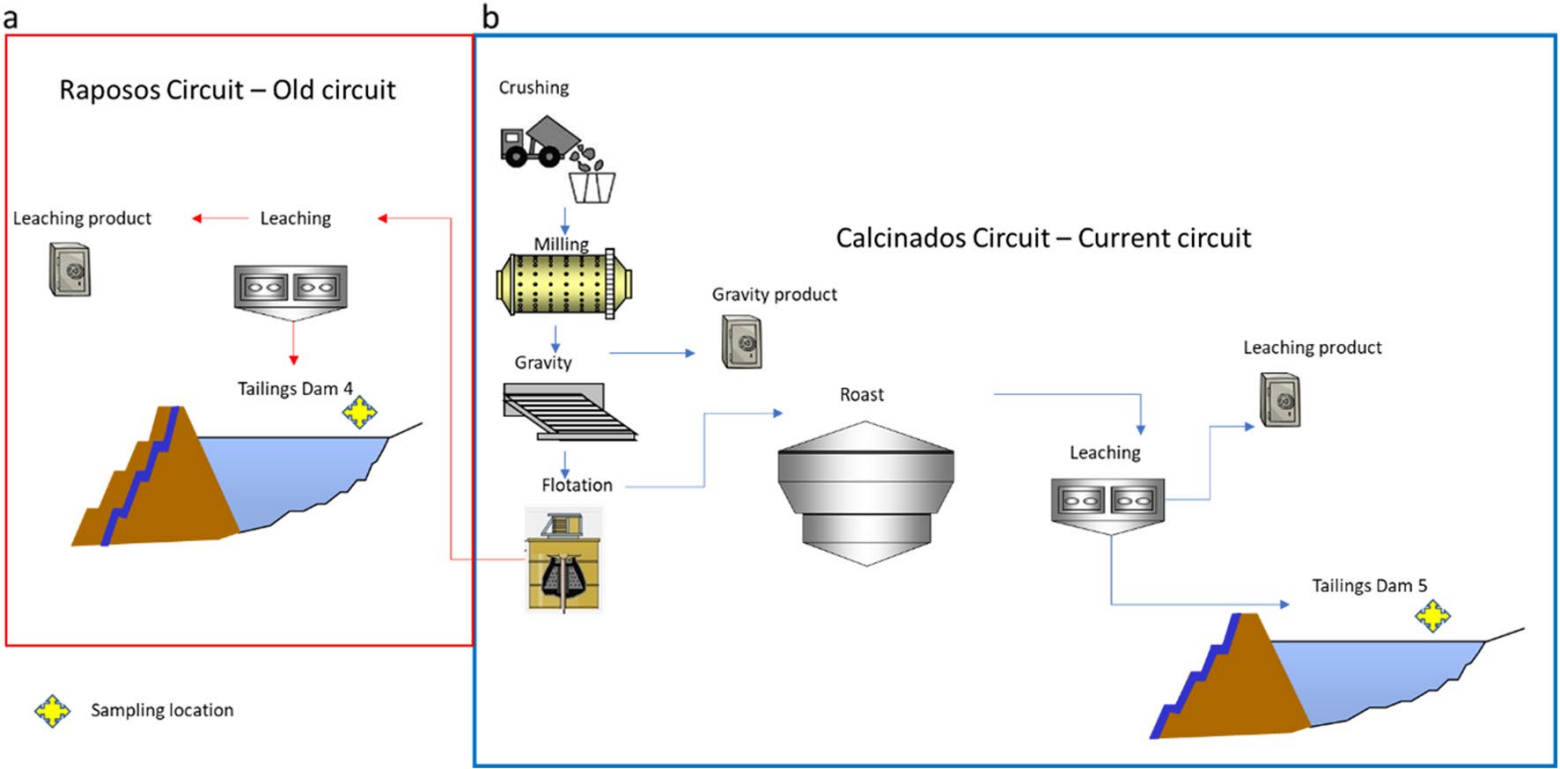
Schematic flowchart of the process that fed the Au extraction dams 4—old circuit (**a**—red line) and 5—current circuit (**b**—blue line) of the Nova Lima Metallurgical Plant.

The Nova Lima plant has been processing sulfide gold ores, no-refractory, for over 30 years. The materials processed at the plant used to be subdivided into two distinct circuits. The old circuit, named as Raposos (Fig. 2a), has achieved 90% Au recovery and the workflow are represented grinding, gravity concentration, conventional leaching and CIP (carbon in leaching), elution, and electro-recovery. This metallurgic plant operated until 1998 with the decommissioning of the Raposos underground mine^9,53,54^. Waste from this circuit was deposited in tailings dam 4 (Fig. 1c).

In the current workflow at the Nova Lima plant, the Au is extracted from the sulfide concentrates through roasting and further leaching with cyanide (Fig. 2b). The tailings are deposited at the dam labelled as 5 (Fig. 1c). The content of Au feed from current mines varies from 5 ppm to 1, depending on each mine ^1^The tailings dams 4 and 5 are downstream, monitored and declared safe according to the national mining agency^5^.

## Materials and methods

### Study design

**Figure 3:**
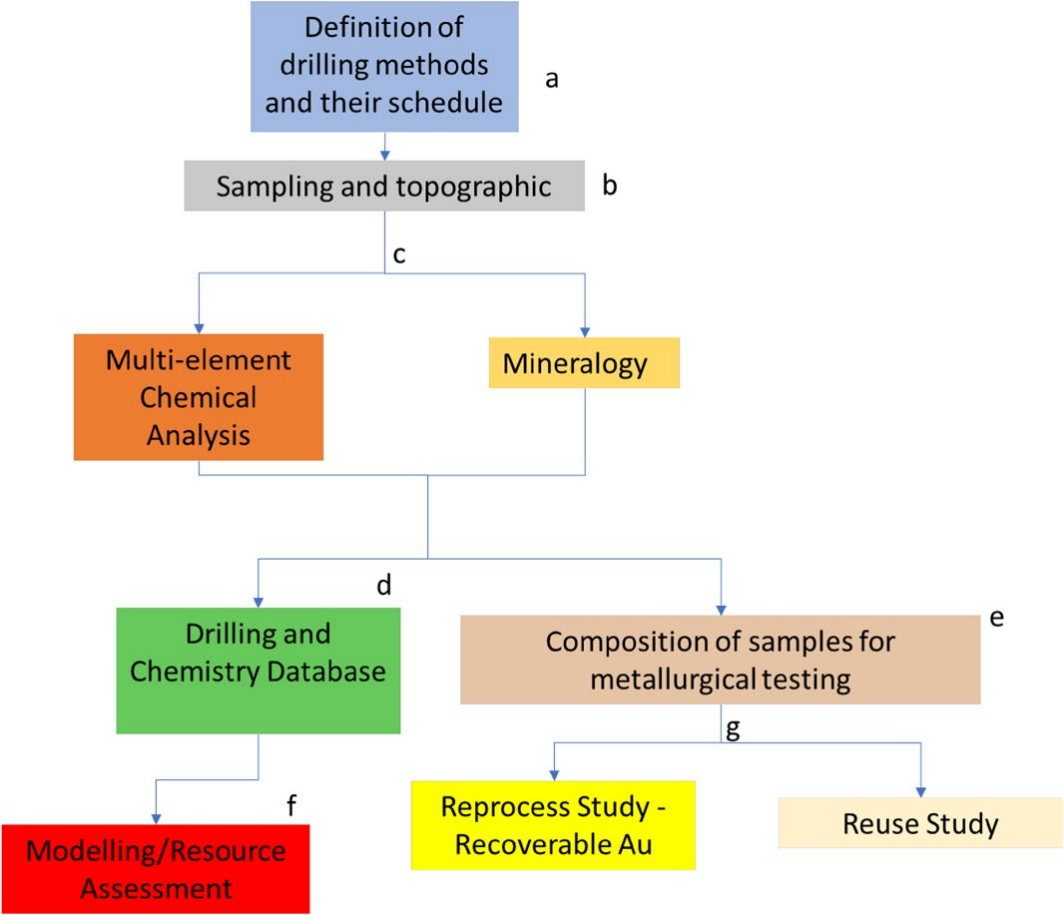
Methodology applied to study tailings from deposits 1 to 5. (**a**) Step of defining the collection methodology for each deposit, (**b**) sampling of deposits and survey of the exact position of each sampled point, (**c**) stage of collecting information on the about chemistry and mineralogy of the samples, (**d**) assembly of a database containing survey and characteristics of each sample, (**e**) 3D modelling and geostatistical analysis of information obtained from sampling, (**f**) composition of samples after 3D modeling for metallurgical tests e, (**g**) study of the potential extraction of Au and reuse of the tailings in its entirety.

The flowchart presented in Fig. 3 illustrates the different stages of this study.

### Sampling

The sampling campaign was carried out during winter and early spring (May–September 2020) when weather conditions vary greatly. Normally, during these months weather conditions are dry and slightly humid. The temperatures ranging from 15 to 25 °C^52^. Depending on the type of deposit, the sampling techniques included percussion, direct push and diamond drilling methods, up to a maximum of 20 m depth, totalizing 1342 m of samples. The methods used, the average depth and the drilling mesh varied according to the sampled deposit. Additional information on the sampling procedures is provided as supplementary material (Fig. S1a,b, Table S1).

1586 samples were collected at 1 m intervals in depth and the position of the samples (hole starting point) were surveyed for exact control of the position of the cores. The samples densities were obtained from those with 100% recovery inside the sampling cylinder of known volume. After weighting, the density was calculated and added to the database^2^.

All samples were transferred to polypropylene bags and kept at a 5 °C until chemical analysis.

### Analytical procedures

#### Chemical analysis

Before chemical analysis, all samples were homogenized and ground to a 2 mm size fraction, quartered and a quarter was ground to a − 37 µm particle size. The coarser fraction was stored for mineralogical analysis and metallurgical tests.

The main chemical analyzes were obtained with inductively coupled plasma mass spectrometry (ICP-MS, PerkinElmer SCIEX, Waltham, Massachusetts, USA). Before analysis, the samples were submitted to acid digestion (nitric acid, hydrogen peroxide, and hydrochloric acid) in the laboratory of AngloGold Ashanti and SGS in Brazil (complementary table S1). Sulfur (S) and carbon (C) concentrations were obtained by infrared detection (LECO, St Joseph, Michigan, USA, detection limit 0.01%). In addition, all Au analyzes were performed by atomic absorption spectroscopy (AAS, Varian, Palo Alto, California, USA—detection limit 0.05 mg/kg) using the fire assay method. All data were processed and validated using the Iogas^tm^-Reflex-64.v 7.2.1 software.

For all analyses, duplicates, blanks and standard reference materials (Si81 from Rocklabs) were included. All controls were obtained within the necessary references and ensured the quality and accuracy of the analyses.

#### Mineralogy

The − 2 mm particle size fraction was used for mineralogical analyses. About 80% of the samples were used in the mineralogical study due to the good recoveries obtained in the sampling. The high number of samples allowed a good representativity of the tailings and enabled identifying variations in and between deposits.

The mineralogy of the samples was obtained though scanning electron microscopy (Scanning Electron Microscopy—SEM-FEI quanta 200) coupled with an energy dispersive spectrometer (EDS) and mineral liberation analyzer software (MLA-FEI). Before the analysis, the samples were prepared in polished section and described under an optical microscope (Leica DM4500 P LED). To produce the MLA equipment database, the main phases were analyzed using a Jeol JXA 8900RL WDS/EDX microprobe. The spectra obtained by wavelength scanning (WDS) entered the MLA software for calibration and subsequent automated analysis. During automated analysis of the obtained data, two modes were used: grain based X-ray mapping (GXMAP) to collect modal information and sparse phase release (SPLDZ) to collect information related to Au-bearing minerals^55^.

Dataview v 3.1.4.686 software was used to process the MLA data as it enables to analyze quantitative mineralogical data obtained through the MLA measurement software. Pixel data is combined with the chemical composition and density of the identified minerals, allowing various analyzes such as modal mineralogy^56^.

#### Gold metallurgical tests

Estimation of the Au extraction potential in the deposits under study was based on the previous geochemical and mineralogical characterization of the samples. Then, samples representing the most enriched zones of the modeled deposits were submitted to two different leaching procedures to define this Au extraction potential.

Within the scope of this work, two protocols were developed for Au extraction in samples ground at 74 µm. The first involved calcination and leaching steps and the second consisted of direct leaching. These two protocols, based on the work of^39^, adapted according to the sample’s mineralogy. According to these authors, finely disseminated gold associations in quartz-sulfide, sulfides of non-ferrous metals, and oxide minerals as hydrogoethite, are major challenges for the industry due to the complexity of extraction. For this type of gold association two types of metallurgical processes are suggested: flotation + calcination + cyanidation and calcination + cyanidation. Therefore, two similar procedures were proposed for the samples of the five study areas (supplementary Fig S2).

### Modelling

Once the database was complete, geostatistical tools were applied to analyze the distribution of elements of economic interest and the contaminants. All models are 3D block model with discretization cell of 5 m × 5 m × 1 m^13,23,57^.

The analysis comprised the assessment of sampling errors, namely sample recovery efficiency, removal of outliers to avoid overestimation of the models (capping), a statistical summary, estimation, and validation. The topography of the deposits was used to define the boundaries of the area to be modelled (Fig. S3). Topographic data used for 3D modeling were obtained using the total station method. The initial position (0 m depth) of all samples was also obtained by this method. The use of surface topography in modeling is important as it influences the calculations of the volume of deposited or stacked material, in addition to being a necessary tool for physical stability in future operations^11,13^. The volumes were defined by the topography limit and the density of the samples (see “Sampling”). Density data was interpolated by block kriging, and each block presented its density value. In this way, we could calculate the model volume. The method was used for all tailings deposits. All data were processed using leapfrog v2021.1 software and datamine studio RM. Principal component analysis (PCA) was performed on 19 geochemical variables to define the primary element associations. This approach put in evidence mineralogical variations within the study areas, important for the interpretation of the source materials and deposition processes of each structure. PCA is a widely used statistical technique to reduce the spatiality of the dataset, decreasing the number of variables by transforming variables that are highly correlated, preserving data variability whenever possible. The transformed variables, referring to the principal components, are a linear function of those of the original data set^13,58,59^. All analyzes were performed in the software Iogas^tm^-Reflex-64.v7.2.1.

To define the spatial patterns of variation of Au concentrations in the tailings deposits, geochemical maps were obtained through ordinary kriging^13,21,22,60,61^ for the previously defined volumes. The identification of Au spatial patterns was critical to identify areas of high contents. The omnidirectional variogram calculated for the geochemical variables was used to obtain the models of spatial variability^62^. The use of variograms with omnidirectional ellipsoid are due to the existent uncertainty in the location of feed sources in the dams and tailings dumps. Based on the model of spatial continuity it was possible to obtain the distribution of Au concentrations for each area.

## Results and discussion

### Mineralogy

A detailed mineralogical characterization of the wastes was performed to obtain the base knowledge necessary to develop extraction procedures suitable to achieve the recoveries that guarantee economic viability. This characterization is of uppermost importance for the proposed aims since the mineralogical phases occurring in the wastes differ from those in the primary ore. Table 2 presents the results of the tailings’ mineralogical characterization deposits under study.

**Table 2:** Means values of the modal mineralogy of the deposits 1 (**N = 130), 2 (N = 207), 3 (N = 400), 4 (N = 229) and 5 (N = 193).

Minerals	Ideal formula	1 (wt%)	2 (wt%)	3 (wt%)	4 (wt%)	5 (wt%)
Quartz	SiO_2_	36.65	31.57	37.83	55.8	15.6
Feldspar group						
Albite	NaAlSi_3_O_8_	2.81	5.33	7.56	0.37	1.5
Anorthite	CaAl_2_Si_2_O_8_	0.010	–	0.030	0.010	0.053
K-feldspar	KAlSi_3_O_8_	1.22	0.120	0.730	0.390	–
Phyllosilicates						
Muscovite group	KAl_3_Si_3_O_10_(OH)_1.9_F_0.1_	27.0	9.08	32.7	112.0	17.1
Oxides						
Fe oxide/hydroxide	Fe_2_O_3_/FeOOH	8.95	–	9.06	8.86	56.8
Rutile	TiO_2_	0.560	0.190	0.600	0.490	0.599
Carbonates						
Ankerite	Ca(Fe,Mg,Mn)(CO_3_)O_2_	0.850	16.8	1.49	11.2	1.00
Siderite	FeCO_3_	8.94	2.92	0.010	7.25	–
Calcite	CaCO_3_	–	0.020	0.230	2.25	0.200
Sulfates						
Gypsum	CaSO_4_ 2H_2_O	–	–	–	0.030	7.00
Sulfides						
Pyrite	Fe^2+^S_2_	0.220	0.310	0.060	0.500	0.002
Pyrrhotite	Fe^2+0.95^S	4.70	0.060	0.148	0.790	0.004
Arsenopyrite	Fe^3+^AsS	1.71	0.520	0.022	0.240	0.056
Chalcopyrite	CaMg(CO_3_)_2_	0.210	0.010	–	–	–
Gersdorffite	NiAsS	–	0.020	–	–	0.010
Covellite	CuS	0.010	–	–	0.070	0.100
Sphalerite	ZnS	–	–	–	0.010	–
Au minerals*						
Native Au	Au > 80%, Ag, Cu, Hg	60	45	2	364	526
Electrum	Au = 80%, Ag = 20%	5	8	1	10	42

*Au particles accounting; **N number of samples.

**Figure 4:**
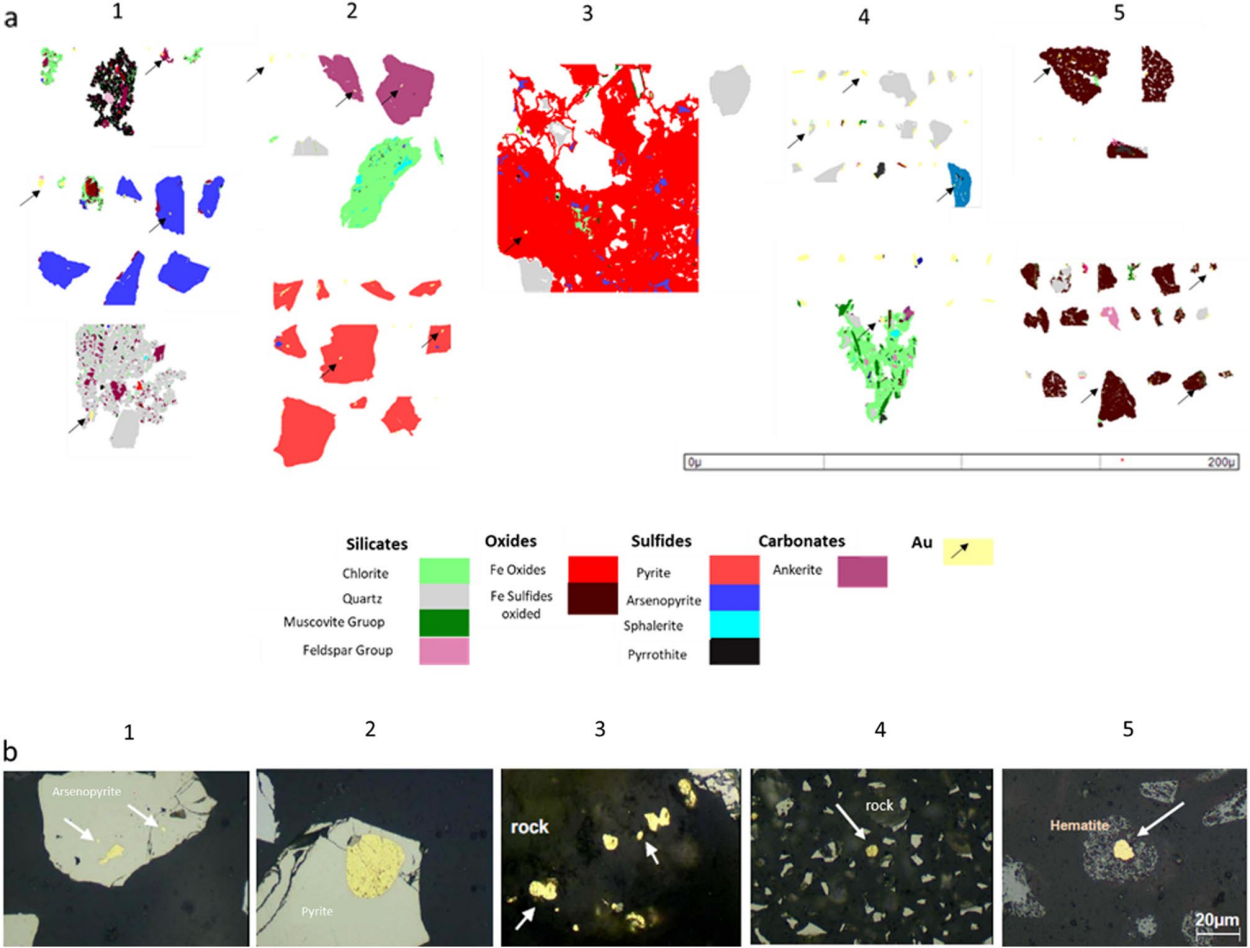
(**a**) False color electron images of Au-bearing particles (yellow and black arrows): Deposit 1—Gold enclosed in pyrrhotite (black), chlorite (green), arsenopyrite (blue) and quartz (gray). Deposit 2: Free and enclosed gold particles in ankerite (purple), chlorite, quartz, and pyrite (dark pink). Deposit 3: Relics of arsenopyrite and gold particles enclosed in iron oxide (red). Deposit 4: Free gold particles and associated in quartz, chlorite, and Deposit 5: Gold particles enclosed in iron oxide and silicates (pink and gray). (**b**) Microphotographs of coarse gold particles associations (reflected light and parallel Nicols) in arsenopyrite (deposit 1), pyrite (deposit 2), rock minerals as quartz, chlorite, muscovite (deposits 3 and 4) and Hematite (deposit 5).

In general, the gangue minerals are similar between the studied deposits, except for dam 5, the dam receiving wastes resulting from calcination and leaching procedures used in the Nova Lima facilities. However, statistically significant differences were observed between deposits 1 and 3, the first mainly composed by carbonates such as ankerite and siderite, and the later dominated by minerals from the feldspar group. The primary silicate is quartz containing Au inclusions, mainly in dams 4 and 3 (Fig. 4).

The primary sulfides and their association with Au express the distinctive mineralogical features between deposits 1, 2, 3, and 4. Pyrrhotite, pyrite, and arsenopyrite are abundant, and their ratios vary between deposits. While pyrrhotite/arsenopyrite are frequently observed in samples from deposit 1, pyrite/arsenopyrite are found in deposit 2. Although in considerably lower amounts relatively to deposit 1, the main sulfide mineral found in deposits 3 and 4 is pyrrhotite. In the sulfides of deposits 1 and 2, Au phases occur as inclusions (Fig. 4b1,b2). The differences in sulfide mineralogy in these deposits may indicate (1) the different sources, that is, wastes originated from mines having distinctive mineral assemblages; (2) different ore processing methods, since dam 4 received flotation wastes (Fig. 2), while the others received wastes from gravimetric concentration and leaching; (3) differences in the kinetics of flotation and leaching processes, since historically the concentration and types of reagents used focused exclusively on the concentration of pyrite, rendering higher concentrations of pyrrhotite in the wastes; (4) different forms of waste storage, since deposits 2 and 4 are dams and deposits 1 and 3 correspond to waste piles. Dam 5 shows a different mineralogical composition, having more than 50% of Fe oxides with small Au inclusions (Fig. 4b5). The presence of high content of oxides in the tailings is due to the different ore processing used in the metallurgical plants that include a roaster to extract Au^9,54^. Comparing with the other deposits, deposit 5 presents the greatest degree of mineralogical transformation between the source and the place of storage. All concentrated sulfides underwent high temperature transformations and were thus calcined, explaining why these Fe oxides are enriched in As, Cu, Ni, and Ag, besides Au (Fig. S4). The presence of gypsum was also observed, probably formed by the addition of reagents such as lime during the calcination process. The other deposits, characteristically, comprise more common minerals because they receive wastes from less aggressive processes such as flotation, gravimetry, and leaching.

In addition to the distinctive mineral associations, the composition of Au grains varies between the deposits. Native Au occurs in all deposits; electrum is more common in dams 4 and 5. In deposits 1 and 2, Au is mainly associated with the sulfides while in deposits 3 and 4 the precious metal occurs in quartz and other silicates, as well as carbonates. In dam 5, Au is mainly associated with iron oxides. These results represent the different sources and treatment processes that originated the wastes deposited in these structures.

#### Chemistry

To evaluated differences in the chemistry of the tailings, concentration of 19 chemical elements were analyzed distance in samples collected at one meter along the core. Table 3 shows a statistical summary for the chemical results obtained for the solid wastes.

**Table 3:** Statistical summary of chemical variables of tails deposits 1 to 5.

Variable	Deposit	N	Mean	S.D	Min	Q1	Median	Q3	Max	Skew
Ag (mg/kg)	1	266	LOD	LOD	LOD	LOD	LOD	LOD	LOD	LOD
	2	162	0.516	0.199	0.230	0.330	0.460	0.690	0.900	0.320
	3	615	0.658	2.21	0.250	0.250	0.250	0.250	13.8	6.03
	4	286	1.51	0.134	1.50	1.50	1.50	1.50	3.00	11.1
	5	230	7.27	3.31	1.00	6.00	8.00	9.00	15.0	− 0.530
Al (%)	1	266	5.24	2.71	0.410	3.12	5.57	7.46	9.70	− 0.340
	2	162	2.94	6.03	0.100	0.460	0.660	3.30	26.3	3.60
	3	615	1.07	0.536	0.233	0.541	1.09	1.53	2.00	0.070
	4	286	3.26	2.17	1.33	2.03	2.50	3.42	11.0	2.12
	5	230	2.92	1.07	0.780	2.28	2.75	3.40	8.46	1.82
As (%)	1	266	0.532	0.322	0.120	0.240	0.470	0.834	1.00	0.280
	2	162	0.634	0.416	0.100	0.200	0.600	0.910	1.35	0.460
	3	615	0.009	0.109	0.100	0.100	1.99	0.0006	1.99	14.8
	4	286	0.550	0.205	0.123	0.417	0.557	0.674	1.00	0.250
	5	230	0.868	0.137	0.281	0.804	0.886	1.00	1.00	− 1.47
Au (mg/kg)	1	266	0.695	0.606	0.115	0.250	0.415	1.05	2.54	1.14
	2	162	1.28	0.819	0.250	0.630	1.16	1.73	3.30	0.940
	3	615	0.362	0.245	0.100	0.150	0.300	0.500	1.19	1.26
	4	286	0.961	0.718	0.100	0.595	0.772	1.19	8.71	5.56
	5	230	2.40	1.06	0.127	1.93	2.35	2.57	9.45	3.70
C (%)	1	266	1.08	1.28	0.100	0.300	0.410	1.40	4.37	1.62
	2	162	2.56	1.76	0.100	0.430	2.45	4.30	4.95	− 0.070
	3	615	2.68	0.124	2.52	2.62	2.70	2.69	3.30	3.70
	4	286	3.60	1.17	0.130	3.48	3.82	4.10	12.1	0.140
	5	230	0.230	0.222	0.100	0.120	0.140	0.180	0.900	2.21
Ca (%)	1	266	0.720	0.919	0.100	0.200	0.400	0.770	4.82	3.15
	2	162	1.35	1.61	0.100	0.170	0.490	3.27	4.46	0.930
	3	615	0.563	0.656	0.132	0.234	0.437	0.677	4.19	4.79
	4	286	4.045	1.86	0.150	3.76	4.26	4.46	16.0	3.03
	5	230	2.70	0.656	0.160	2.36	2.61	2.92	5.75	0.460
Cu %	1	266	0.344	0.249	0.100	0.152	0.230	0.492	0.900	1.06
	2	162	0.487	0.250	0.110	0.200	0.500	0.700	0.800	− 0.380
	3	615	0.464	0.271	0.100	0.188	0.432	0.670	0.980	0.410
	4	286	0.267	0.184	0.100	0.172	0.213	0.260	0.990	2.31
	5	230	0.550	0.365	0.111	0.129	0.683	0.890	0.998	− 0.180
Fe (%)	1	266	8.88	5.44	1.48	3.47	7.77	15.0	15.0	0.010
	2	162	8.54	3.24	1.24	6.20	8.80	9.95	14.2	− 0.220
	3	615	0.003	0.006	0.005	0.005	0.080	0.005	0.080	5.95
	4	286	12.6	3.22	1.00	12.5	13.6	15.0	15.0	− 2.02
	5	230	8.02	13.4	4.00	4.00	4.00	4.00	67.0	3.33
Mg (%)	1	266	1.08	1.28	0.100	0.305	0.550	1.38	4.80	1.76
	2	162	1.90	1.07	0.300	0.900	2.12	2.76	3.35	− 0.270
	3	615	0.641	0.558	0.129	0.283	0.538	0.844	3.22	2.96
	4	286	2.201	0.918	0.130	1.58	2.49	2.84	3.90	− 0.610
	5	230	0.597	0.1435	0.170	0.515	0.600	0.700	0.930	− 0.370
Mn (%)	1	266	0.385	0.269	0.100	0.140	0.300	0.600	0.900	0.610
	2	162	0.331	0.170	0.100	0.184	0.322	0.415	0.733	0.840
	3	615	0.418	0.278	0.111	0.160	0.381	0.649	0.940	0.570
	4	286	0.295	0.068	0.100	0.280	0.300	0.320	0.800	1.92
	5	230	0.434	0.370	0.100	0.100	0.130	0.900	0.900	0.300
Na (%)	1	266	0.642	0.491	0.100	0.245	0.430	0.960	1.81	0.850
	2	162	0.698	0.421	0.110	0.430	0.600	0.900	1.90	1.33
	3	615	0.201	0.144	0.100	0.132	0.158	0.230	0.921	3.93
	4	286	0.609	0.273	0.120	0.340	0.680	0.8300	1.6	− 0.120
	5	230	0.233	0.100	0.100	0.175	0.220	0.260	0.900	3.20
Ni (mg/kg)	1	266	76.4	57.3	1.00	36.5	61.0	112	298	1.26
	2	162	193	109	23.0	157	168	233	547	1.75
	3	615	61.6	52.0	0.500	34.0	48.3	85.6	271	2.14
	4	286	80.2	50.1	1.00	54.0	81.0	99.0	243	0.670
	5	230	425	199	3.00	326	497	559	744	− 0.890
P (%)	1	266	0.333	0.142	0.100	0.200	0.300	0.400	0.800	0.550
	2	162	0.397	0.145	0.250	0.330	0.380	0.410	0.950	3.30
	3	615	0.350	0.180	0.132	0.230	0.291	0.396	0.876	1.66
	4	286	0.360	0.0978	0.100	0.300	0.300	0.400	0.800	1.96
	5	230	0.459	0.073	0.300	0.400	0.500	0.500	0.700	0.680
Pb (mg/kg)	1	266	29.9	92.6	1.00	9.00	14.0	21.5	749	7.57
	2	162	148	146	7.00	22.1	92.6	297	429	0.670
	3	615	26.7	90.1	1.50	3.90	10.5	16.6	564	6.04
	4	286	41.3	23.6	1.00	27.3	42.5	56.0	124	0.170
	5	230	197	106	2.00	142	231	267	383	− 0.600
Sc (mg/kg)	1	266	8.25	6.20	1.00	2.50	7.00	10.0	27.0	1.17
	2	162	14.4	7.27	1.50	9.40	13.1	17.7	28.4	0.180
	3	615	4.18	1.58	1.60	4.00	4.00	4.00	13.4	5.49
	4	286	8.26	5.200	1.00	6.00	7.00	9.00	26.0	1.99
	5	230	7.74	4.16	1.00	6.00	8.00	11.0	22.0	− 0.010
Ti (%)	1	266	0.262	0.185	0.100	0.155	0.200	0.295	0.800	2.13
	2	162	0.459	0.299	0.110	0.180	0.510	0.750	0.970	0.230
	3	615	0.417	0.261	0.100	0.185	0.372	0.628	0.917	0.480
	4	286	0.498	0.185	0.100	0.400	0.500	0.600	0.900	− 0.460
	5	230	0.538	0.186	0.100	0.400	0.500	0.700	0.900	− 0.280
Zn (mg/kg)	1	266	25.2	80.6	1.00	31.0	58.0	112	199	0.670
	2	162	11.4	34.7	6.00	46.0	94.0	127	167	− 0.080
	3	615	11.3	42.2	2.20	30.6	53.6	123	368	2.03
	4	286	162	311	1.00	43.0	86.0	148	3419	7.21
	5	230	1872	1796	18	499	2778	3641	6187	− 0.080

*LOD *bellow detection limit*.*

In general, the 19 chemical variables present dissimilar distributions that are expressed by the variable skewness coefficients, as can be observed in Table 3. Heavily skewed distributions are expected due to the presence of outlier values (supplementary Fig. S5). Asymmetric distributions represent mixtures of populations, and can be defined due to multiple and different sources or processes^63^. The data presented in Table 3 show that mean Fe concentration, in addition to Cu, Zn, Ag, Pb, and Ni, are higher in deposit 5. This can be explained by the presence of Fe oxides containing these elements (Table 2; Fig. S4). Here, the C concentrations are lower than those of the other deposits.

Deposits 1, 4, and 5 are marked by the highest average of S, Al, and Ca. Sulfur concentrations can be explained by the presence of sulfides, while Al is related to the presence of aluminum silicates such as muscovite and chlorite (Table 3). Deposits 3 and 2 are depleted in S and Ag. The Sc content is relevant for deposit 2 and should be further investigated in future work.

Average Au concentrations range, in general, from 0.1 to 2 mg/kg (Table 3). The highest average was found in deposit 5, followed by deposits 4 and 1. The tailings from area 3 show the lowest average concentration, indicating that tailings with lower Au content were accumulated in this deposit. This result is supported by the mineralogical study, which indicated a larger number of Au grains in samples from deposit 5 than deposit 3.

**Figure 5:**
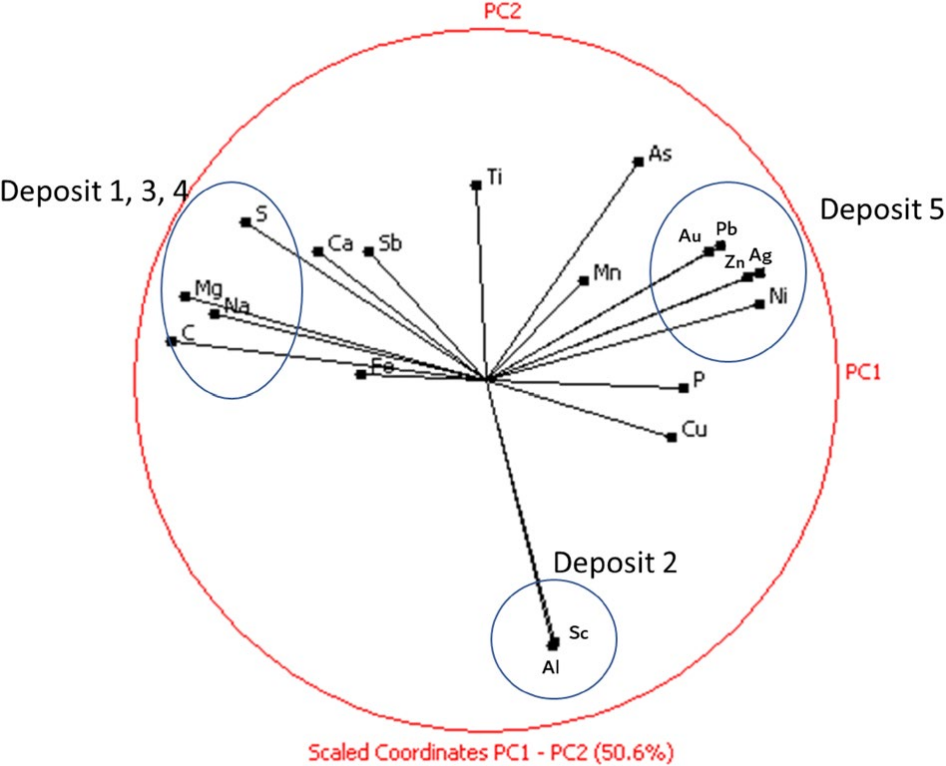
Variables projections on the first factorial plane (PC1/PC2). This plane accounts for about 50% of the total explained variance of the dataset. The blue circles highlight the clusters obtained by the PCA analysis.

Although As is one of the main contaminants, it can also be an element of economic interest^10^. In these deposits, the geochemical pattern of As is similar to that of Au, and the element is more abundant in deposits 5, 4, and 1. From Fig. 5 and Table 3, the relationship of this element with Au can be explained by the associations found for the Au phases, most commonly with As-Fe containing sulfides in deposits 1 and 4.

The Mn contents are higher in deposit 4, probably due to the presence of carbonates^54^. Although this element is not as hazardous as As, Mn levels must be taken into account in the context of contamination^64^.

The PCA performed for the dataset resulting from the chemical analysis of the tailings collected from the different deposits shows the geometrical relationships between the geochemical variables. About 50% of the variability in the data is explained by the two first principal components (Fig. 5). PCA analysis has identified three distinct groups of geochemical variables. The first comprises elements such as Pb, Ni, Au, Ag, Zn, and Cu. The second group encloses S, Ca, C, and Mg. Comparing these results with those of the mineralogy (Table 2) and geochemistry (Table 3), it is likely that variables in the first group represent the deposit 5 while variables in group 2 represent deposits 1, 3, and 4 (Fig. 5). The Sc-Al association possibly indicates deposit 2, which presents the most elevated Sc concentrations (Table 3).

Therefore, the distinctive geochemical patterns clearly indicate different characteristics for deposits 2 and 5 and common features for deposits 1, 3, and 4 (Fig. 5, Fig. S6). Such detailed information is essential to assess the metallurgical potential for reuse and the impacts associated with the disposal of these tailings^6,9^.

### 3D modelling of Au contents

**Figure 6:**
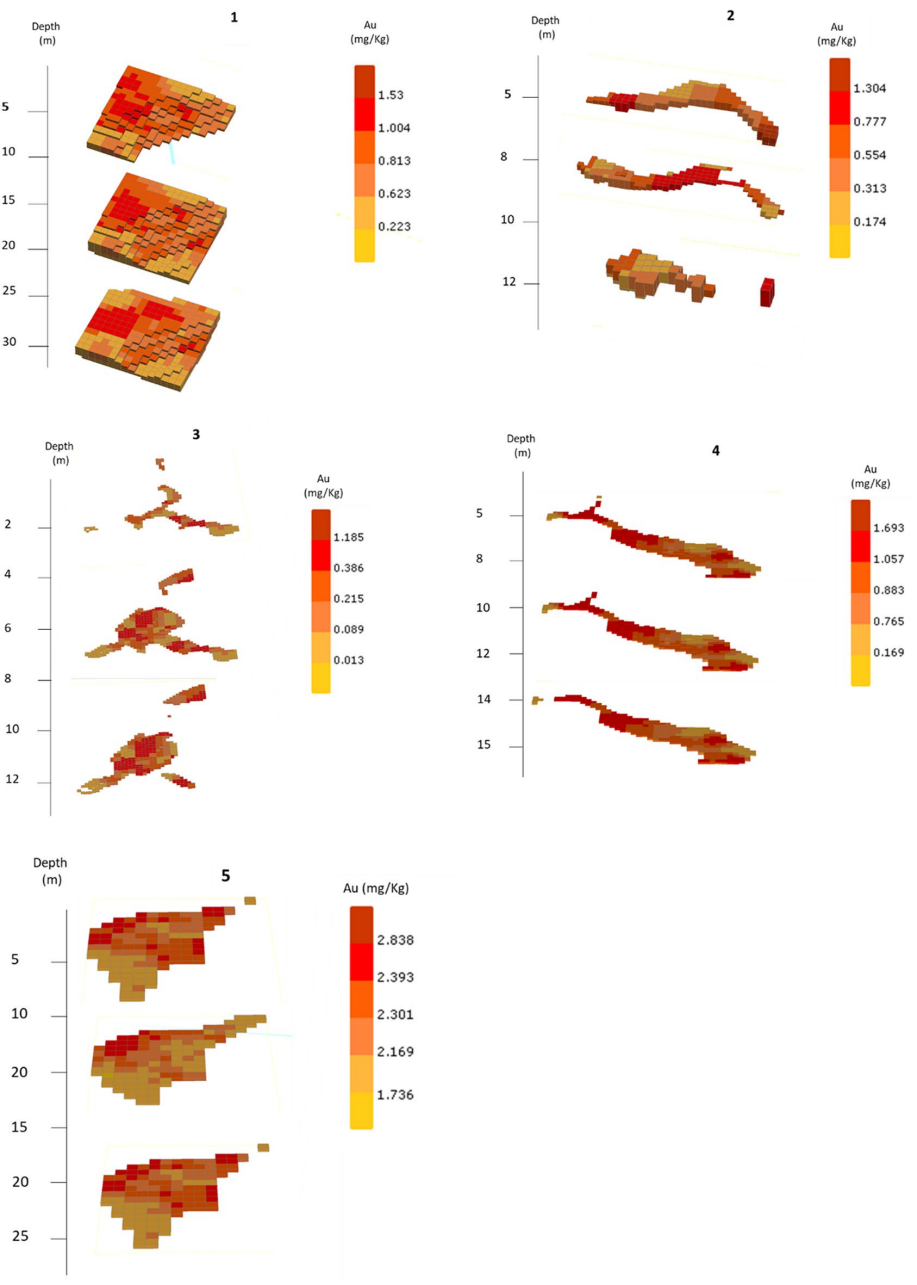
Spatial distribution of Au grades from 3D model by elevation and deposits 1 to 5.

Besides to the mineralogical and geochemical characteristics of the deposits, the size and shape of the mineral masses are also important factors to consider when assessing the potential for the valorization of the wastes. Therefore, for the Au concentrations, a 3D spatial continuity model was defined for the five deposits considering the heterogeneity study of each one individually. For each deposit, a variogram was built that considers the individuality of the spatial continuity of the distribution of the Au concentration.3D maps obtained by ordinary kriging are presented in Fig. 6. In these maps, the concentration intervals correspond to the minimum, the quartiles, and the maximum value of the kriged blocks. From the maps, it is possible to identify the distributions of Au concentrations within the deposit and target enrichment areas that can be used in feasibility analysis and reserves estimations.

**Figure 7:**
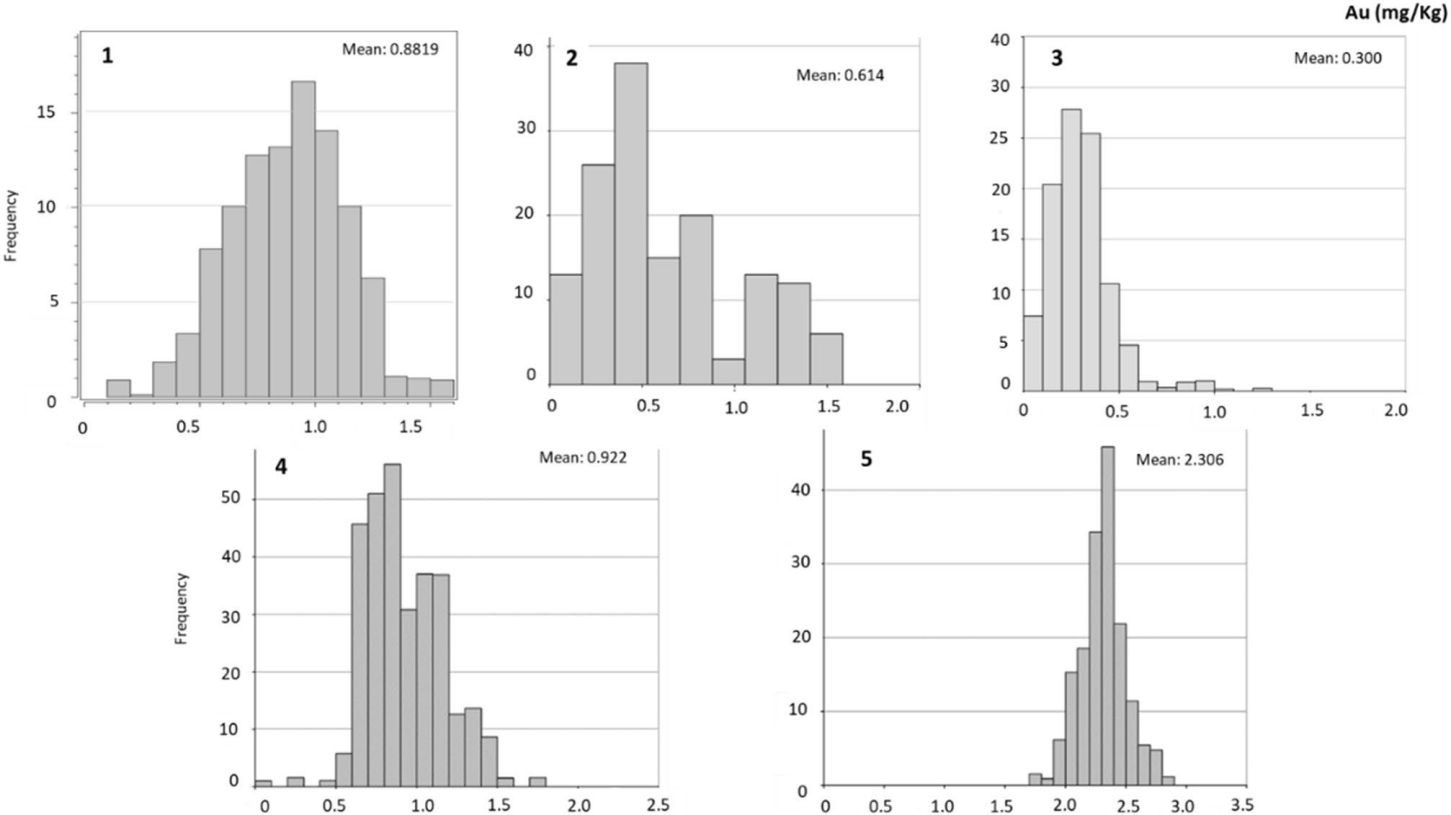
Distribution of Au contents in the block from 3D model for deposits 1 to 5.

Figure 7 shows histograms with Au distribution in the blocks of the 3D model of deposits 1–5. Table 4 shows the mean estimated concentrations of Au, as well as the tonnages and ounces for the five deposits under study.

**Table 4:** Estimation resources of Au for deposit 1 to 5.

Structure	Tonnes (Mt)	Au (mg/kg)	Onces (Moz)
1	501	0.88	14
2	7	0.61	0.179
3	413	0.30	4
4	3350	0.92	100
5	2048	2.30	150

For deposit 1, the ounces obtained by this modeling represent 14 Moz (Table 4). Enrichment areas with Au grades above 1 mg/kg are observed in the northwest portion of the area. Concentrations above 1 mg/kg also occur in blocks from 15 m deep forward (Fig. 6). The blocks show an average concentration close to 1 mg/kg (Fig. 7), indicating potentially interesting values for the waste volume under study.

In deposit 2, it is observed a limited continuity in Au contents with depth. Small enrichment zones occur at the east boundaries of the deposit for the first 5 m depth and in the center for the next 10 m (Fig. 6). The highest frequency obtained for these blocks is 0.5 mg/kg (Fig. 7), although several blocks have contents above 1 mg/kg, which are regarded as enrichment areas. Nevertheless, the amount of Au contained in this deposit is relatively low, represented only by 0.179 Moz (Table 4).

Deposit 3, a stockpile, is less attractive, as the enrichment zones show limited spatial continuity. The random vertical and lateral distribution of these relatively high Au-content areas decreases the potential for valuation of these wastes. The histogram of Fig. 7 also shows most blocks having concentrations below 0.5 mg/kg. Therefore, the Au contained in this deposit is very low (Table 4).

In the deposit 4, high content blocks (Au concentrations equal to 0.8 mg/kg and 1.0 mg/kg) show considerable spatial continuity with depth (Figs. 6, 7). Low grades are observed at the northeast boundaries, but in terms of representation and continuity may be considered negligible since the Au content is 100 Moz (Table 4).

In deposit 5, the Au concentrations increase in the last 40 m depth (Figs. 6, 7). This deposit has the higher Au concentration, reaching 150 Moz (Table 4).

Therefore, based on the estimates, Au contents range from 0.3 to 2 mg/kg. The highest grades (average = 2.30 mg/kg) are obtained for deposit 5. Tailings 3 presents the lowest Au grade, with an average estimate of 0.30 mg/kg. Deposits 1 and 4 show average Au grades that are similar and range from 0.88 to 0.92 mg/kg. Finally, estimated value for deposit 2 is 0.61 mg/kg for the mean Au content.

The results suggest that the deposits 4 and 5 have a high potential for Au recovery and reuse compared to other mines around the world that are described in many research^11,53,65^. Deposits 3 and 2 show lower potential for valuation, as the estimates indicate low metal contents and smaller volumes.

It is important to note that, in addition to Au contents, a range of other factors influence the potential for valuation. The efficiency of metallurgical Au recovery, available technologies, metal price, OPEX/CAPEX costs, and socio-economic situation of the region, are among the most important constraints^13^.

### Au metallurgical tests

According to the results of the 3D models (Table 4), the distribution of Au showed, in general, a potential for extracting this element. Therefore, new information is necessary, namely on the recovery efficiency, to properly assess this potential. Thus, two extraction procedures (Supplementary Fig. S2) were developed based on the mineralogical and geochemical characteristics described earlier for the different tailings.

To test the two procedures, composite samples were obtained from the enrichment zones identified in the 3D models (Fig. 6). In the first procedure, the samples were ground at 74 µm, calcined at 700 °C, and leached with cyanide (concentration 2000 mg/kg) and lime (concentration 2090 mg/kg). In procedure 2, the samples were leached in the same conditions as in scenario 1, but without calcination stage.

The Au recovery (%) obtained for each deposit are presented in Table 5. The results indicated that Au recovery potentials vary with the procedure used and between deposits (Table 5). The recoveries obtained for deposit 5 are low, especially with procedure 1.

**Table 5:** Summary of results of metallurgical tests for reuse of Au.

Deposit	Feed grade (mg/kg)	Au mineralogical association	Procedure 1 (calcine + leach- in 74 µm %Au recovery)	Procedure 2 (leach in 74 µm-% Au recovery)
1	1.16	Au in sulfides and quartz	78.5	64.0
2	1.05	Au in sulfides and quartz	77.7	24.1
3	0.60	Au in sulfides and quartz	53.4	93.0
4	0.87	Au in sulfides and quartz	79.7	50.7
5	2.40	Au in Fe oxides	0.600	32.2

Deposits 1, 2, and 4 showed better results when submitted to calcination followed by leaching (procedure 1). In these deposits, Au is associated with sulfides such as arsenopyrite, pyrite, and pyrrhotite, and the calcination effectively liberate the enclosed Au-particles^9,39^. In addition, an important amount of Au associated with quartz was partially stripped in the leaching stage of procedure 1.

Although dam 5 has better estimated Au grades (Table 4), it was challenging to extract Au using the proposed procedures. Based in mineralogy data, the previous calcination processes produced secondary mineral phases that are extremely resistant, and the Au inclusions are tightly bound to the iron oxides (Fig. 4, Fig. S4). the results indicate that when the wastes underwent calcination, a second calcination stage decreases the efficiency of the extraction.

More than 90% of Au were recovered from deposit 3 samples when subjected to procedure 2 (74 µm leaching), which indicates a high potential to leach Au associated to silicate minerals by the acid solutions. However, as referred, technological challenges are foreseen for the recovery of deposit 5, the one with the highest content. This fact is probably due to the form of occurrence of Au in the Fe oxides (Fig. 4). Gold is extremely fine-grained and is entrapped in the pores of the Fe-oxides formed during the calcination stage of the metallurgical treatment of the Au-ores. Therefore, the mineralogical and textural features of the mine wastes are critical factors for recovery.

Nevertheless, the extraction of these wastes seems feasible and has the potential for valuation. However, together with other variables, it must be carefully evaluated when deciding the destination of these deposits.

#### Potential for generating other products

There is a need to generate and disseminate new technologies, especially the so-called clean and sustainable technologies. These must respond to the great challenges of the mining sector, such as minimizing environmental impact and maximizing social satisfaction^66,67^. Although this work has focused on the recovery of Au, the potential for other applications and use of these tailings should be considered. Therefore, for the five deposits under study, other valuation potentials are also suggested. Important for this evaluation were the mineralogy (Table 2) and the geochemical patterns previously identified (Fig. 6). Table S2 shows other proposals for the potential valuation of these tailings.

In general, some method of separation, e.g. triboelectrostatic^68^, is efficient to concentrate and liberate sulfides for better extraction of Au. Based on the described mineralogy, the developed methodologies for Au extraction may potentially be used to generate other valuable resources such as vapor generated energy, iron, and gypsum. Furthermore, silicate minerals could form a dry sand for use in construction. In some cases, such as deposits 5 and 4, Fe oxides can also be concentrated by magnetic separation.

In addition, other potential resources can also be investigated. This may be the case of As and Sb, which can be tested to generate a new product, thereby decreasing the potential for contamination. The vitrification of As and Sb has great potential for application in these types of materials^37^.

Also, studies of the use of these materials as rock meal and fertilizers can be of great interest due to the K and Al concentrations^69^.

## Conclusions

In this work, gold mining tailings were studied in order to identify their value through the use of characterization and geostatistical tools. The use of these tools identified Au as a valuable element and generated motivation for paths that avoid undesirable disposal of waste and large-scale environmental impacts. The result of the characterization and modeling showed that Au is the most interesting metal in terms of resources with concentration like low-grade mines currently in operation. Interesting Au enrichment zones were identified in the deposits 1–5 through 3D models. Deposits 4 and 5 are more appealing since they present higher Au concentrations and more homogeneous spatial distributions. In general, wastes discarded in dams show higher valuation potential than waste piles, mainly to the higher lateral and vertical continuity of the Au-contents.

The metallurgical tests show good recoveries for Au. Deposits 1, 2 and 4 presented above 70% for procedure 1, while the au from samples from deposit 3 was better extracted in procedure 2. Even with higher levels of Au, deposit 5 showed low extraction potential, demonstrating a challenge technology for the reuse of Au from this deposit.

Furthermore, in addition to Au, some elements like As, Sc, Mg, Ti, and Si that are in the list of critical elements of the EU, may have potential economic value, indicating the need for future works.

In the present study, through the combination of statistical, mineralogical and geochemical tools, it was possible to assess the potential for valuation of the mining wastes. This study, thus, adds new knowledge contributing to the sustainable development of the mineral sector, towards a future where waste becomes a product and that the generation of zero tailings becomes a reality in this sector.

## Supplementary Information

Supplementary Information.

## Data Availability

The datasets used and/or analysed during the current study available from the corresponding author on reasonable request.
