# Supplementary material for: Geochemistry and mineralogy of auriferous tailings deposits and their potential for reuse in Nova Lima Region, Brazil
Source: Sci Rep. 2023 Mar 16;13:4339. doi: 10.1038/s41598-023-31133-6 (PMC10020576; doi:10.1038/s41598-023-31133-6)
Supplement: Supplementary file 1 — Supplementary Information. [file 41598_2023_31133_MOESM1_ESM.docx]

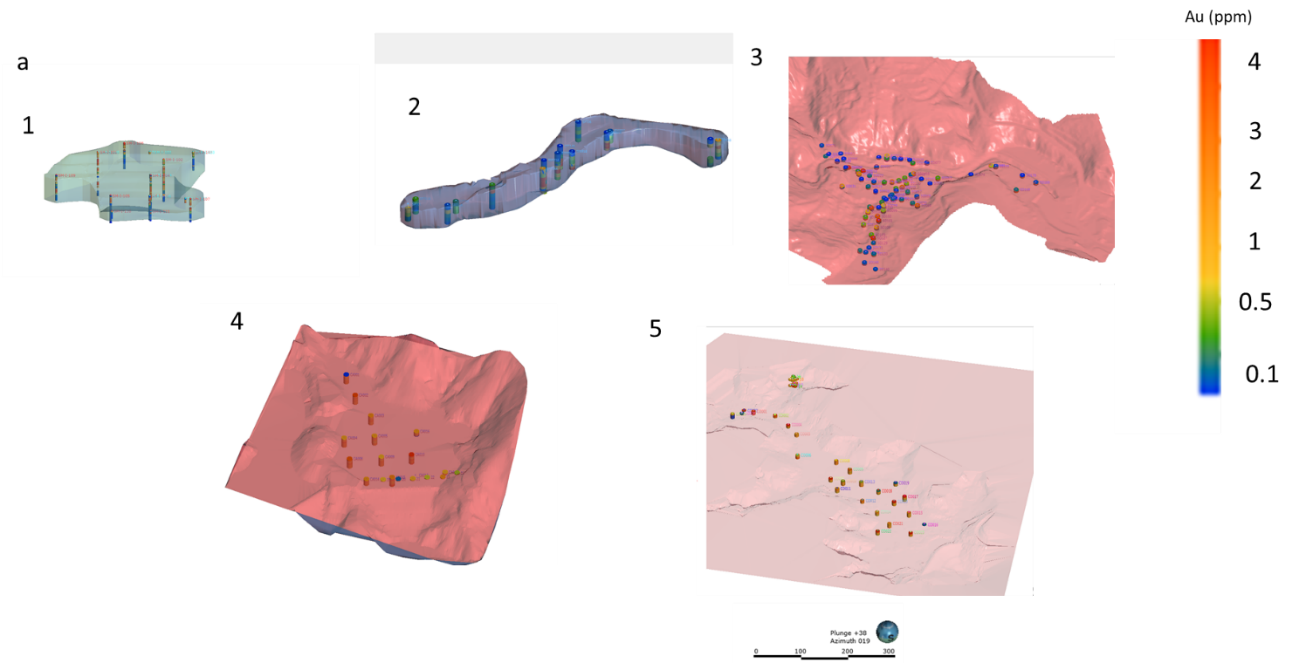


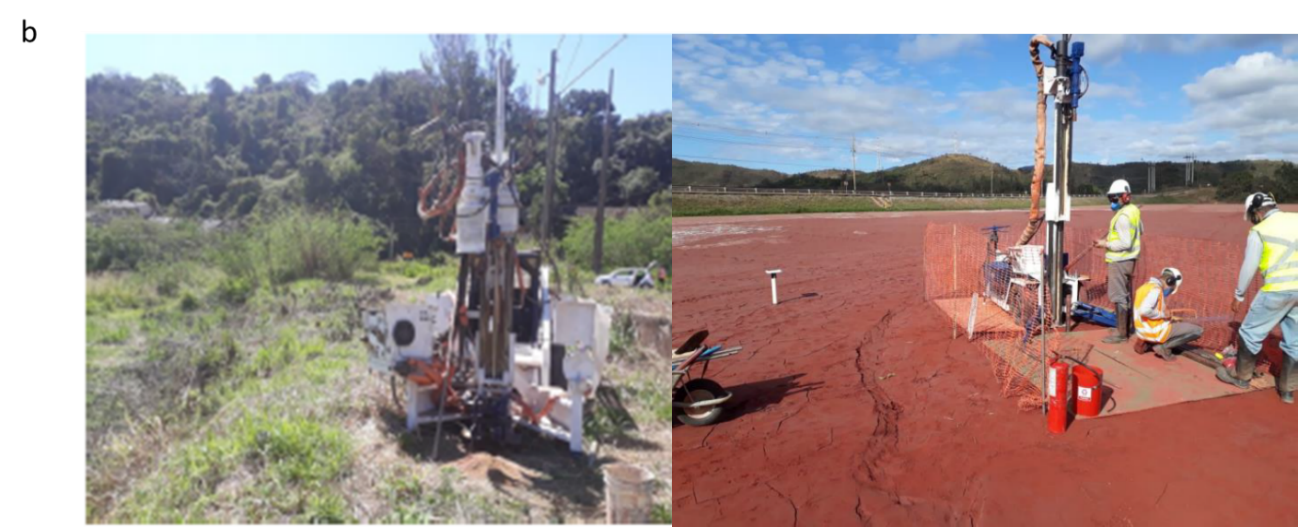


Fig S1. a. Collection points of structures 1 to 5 and b. Instrument images and collections in dry deposits (above) and dam (below)

Table S 1. Sampling Method by Tailings Deposit

| Area | Sampling Method | Depth (~m) | Samples Numbers | Analysis |
| --- | --- | --- | --- | --- |
| 1 | Percussion+Diamond Probing | 15.88 | 266 | ICP, Fire Assay, PSD, Mineralogy and Metallurgical Tests |
| 2 | Percussion Probing | 4.43 | 162 |  |
| 3 | Direct push | 4.0 | 615 |  |
| 4 | Direct push | 6.70 | 286 |  |
| 5 | Direct push | 12.50 | 257 |  |


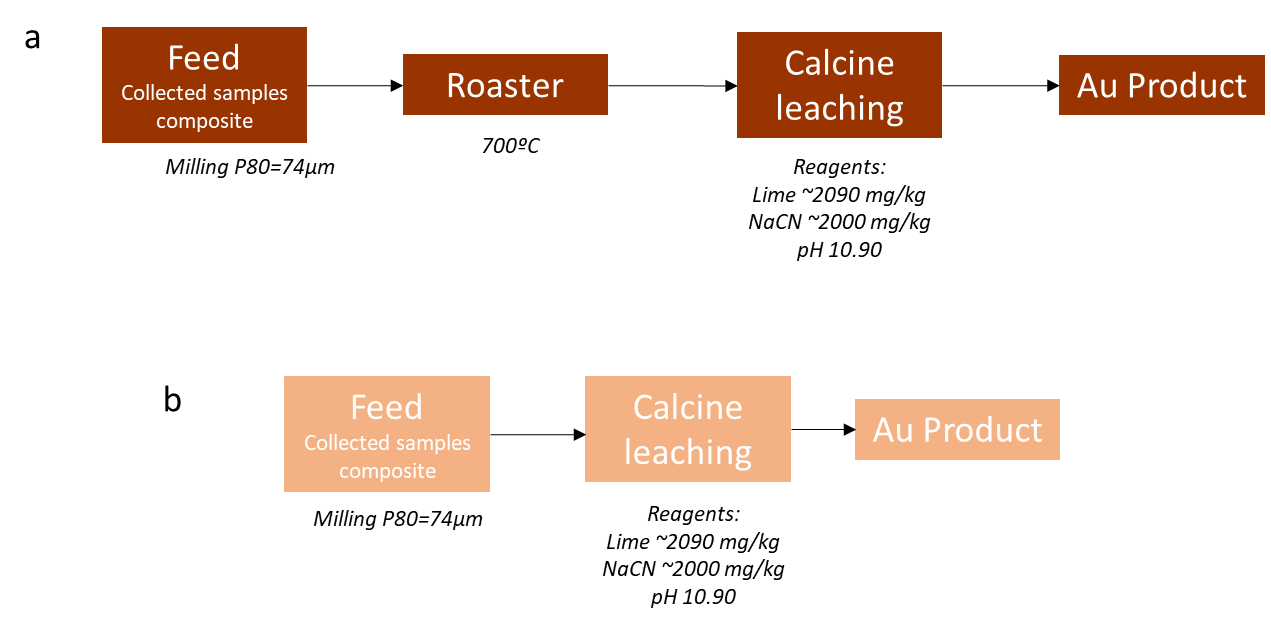


Fig S2. a. Workflow of Au Metallurgical Procedure 1 (a) and 2 (b)


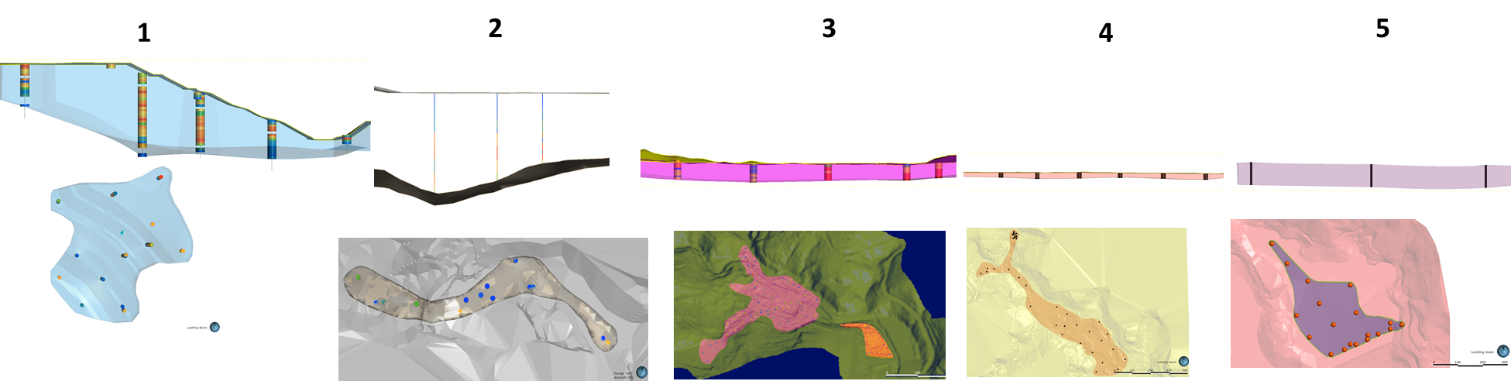


Fig S3. Topography limits of 3D modeled by areas 1 to 5


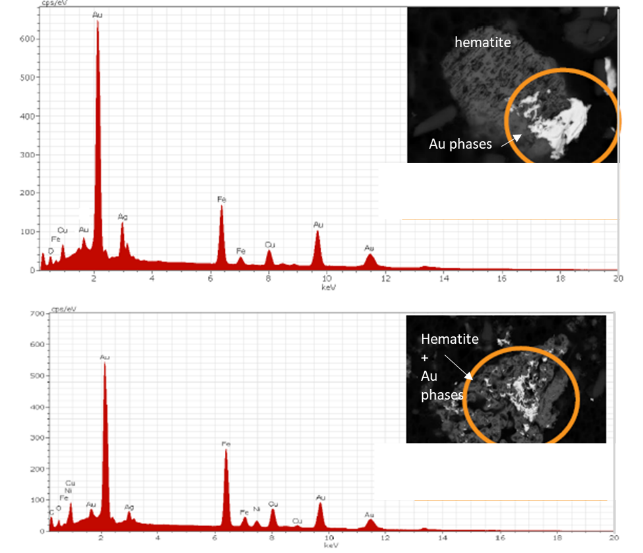


Fig S4. EDS spectra of Fe oxides and Au phases containing elements such as Cu, Ni, Ag, As from deposit 5.


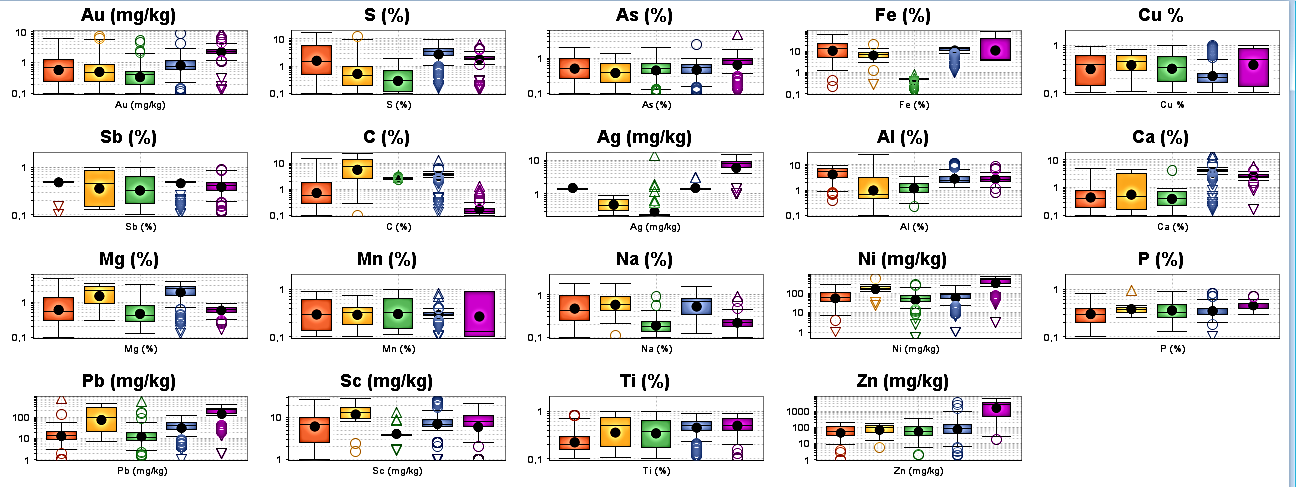


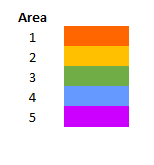


Fig S5. Box plots for 19 elements-based on dataset collected

Table S2. Summary of the main potential uses of the studied samples

| Struture | Product | Au recovery | Reuse potential 1 | Reuse potential 2 | Reuse potential 3 |
| --- | --- | --- | --- | --- | --- |
| 1 | Tailings pile | 78.5 | As vitrification | sand, filler, cement | - |
| 2 | Tailings Dam | 77.7 | As vitrification  Sr recovery | sand, filler, cement | plaster |
| 3 | Tailings pile | 93.0 | As vitrification | sand, filler, cement | - |
| 4 | Tailings Dam | 79.7 | As vitrification | sand, filler, cement | Fertilizers, rock meals |
| 5 | Tailings Dam | 32.2 | As vitrification Fe recovery | - | Fertilizers, rock meals |
